# On Blood-Letting in Apoplexy

**Published:** 1863-10

**Authors:** Thomas K. Chambers

**Affiliations:** Physician to St. Mary’s Hospital, &c.


### MISCELLANEOUS.

### ON BLOOD-LETTING IN APOPLEXY.

BY DR. THOMAS K. CHAMBERS, PHYSICIAN TO ST. MARY'S HOSPITAL, &C.

[Perhaps no question is more serious than that regarding the propriety of taking blood in cases of coma, apoplexy, and paralysis. Delay may be irretrievable; yet a false step may be equally fatal.]

In the first place, let us clearly understand what is the *modus operandi* of the means you design to employ, and then what morbid conditions it can and what it cannot relieve.

The two most marked immediate effects of blood-letting are, diminution of the force of the heart, and contraction of the area of the blood vessels. That these are the most important and most direct of the resulting phenomena, is shown by their being in direct proportion to the dose; the more rapid and copious the detraction of blood, the more certainly they follow. Both natural and artificial hemorrhage thus produce, first syncope, or failure of heart-power; and then a slackening and arrest of the blood-stream by contraction of the vessels.

What now are the morbid conditions in apoplexy which such results as these are likely to benefit? I am not going to enumerate all the causes of apoplexy, because such a recapitulation would be very unsuited for the purpose of the present lecture, and, if they came into your head at the time you were in presence of your patient, would serve rather to confuse than to clear your ideas. A most practical thing to do will be to divide them into three heads with reference to the treatment to be pursued; and I think you will find them all capable of being classified under *destruction* of the nerve-fibres, *compression* of the same by a *solid*, and *compression by a fluid* substance.

Now, if the nerve-fibres be destroyed, as is the case, for example, in what appears after death as a softening of the cerebral substance, it seems unadvisable to take away the pabulum of nutrition; for you would be diminishing the power of the remaining brain-tissue to take the place, as far as it can, of that which is irretrievably lost. Nor are the immediate effects of blood-letting likely to be advantageous; for in these cases the force of the heart is not morbidly augmented, and the blood-stream is in general rather deficient in quantity already. We shall probably be only adding to the disease by substracting blood. When, then, we have reason to think from the previous history that there is old disease leading to softening of the cerebral substance, we should abstain from bleeding altogether. If, for instance, there have been before the present attack a state of mental excitement, or severe headache, or feverishness; or if the gait has been unsteady, the speech thick and stammering; if there have been ear ache, or a purulent discharge from the auditory meatus; if previous to the apoplexy, there have been convulsions; if the patient be scrofulous, or of a scrofulous family, we must take all these circumstances as warnings against the lancet—altogether, probably, but certainly against its immediate use.

Supposing, however, that we have no evidence of such a diseased state of the cerebral substance, and can allow ourselves to conjecture that the nerve-fibres are more compressed than permanently disorganized, then we have further to consider whether the compressing substance is *solid* or *fluid*. By a solid likely to cause apoplectic paralysis, I refer to a clot of blood which has been thrown out long enough to become entirely coagulated. This is the only solid sufficiently common as a cause for us to consider. Is it likely you have to deal with anything of this sort? Is there in the patient's brain before you at the moment a clot of blood such as you see put up in bottles in the museum, and often also in the dead house? Do not be in too great a hurry to conclude so at once. Four days ago you saw on the dissecting-table the corpse of a man who more than thirty-six hours previously had fallen down stairs, and was supposed to have fractured his skull; his cranium was uninjured, the fall was apoplectic, and there was a quantity of fluid blood pressing upon the brain. You see by such instances as these that blood extravasated from the vessels inside the body does not coagulate immediately, as it does when drawn from a vein; it remains long, and may remain much longer than it did in that case, in a liquid state. It takes, not minutes, not hours, but I may really say days, to become solid. When the apoplexy or paralysis has lasted for days, then indeed, but not till then, you may conclude that a clot has formed; and then, and not till then, may you pronounce that the force of the heart's action will not increase the oozing of blood into the focus of pressure. After that—after you have a solid, and not a fluid pressure, to deal with—I am willing to concede, and anxious to impress upon you, that abstraction of blood is useless and hurtful; but till then I believe its immediate influence to be a beneficial one, and in the right direction. Therefore do not conclude that a clot is formed, or that bleeding is for that reason useless, till at least twenty-four hours are passed after the fit.

Supposing that a fluid has to be dealt with, you are perhaps anxious to know of what nature it is; and, in point of fact, I find that I am constantly asked by students, when they see a case of apoplexy in the wards, whether it is serous or sanguineous apoplexy. I say to you now, what I always say then, that there is no possibility of certainly distinguishing them; and that, fortunately, it is of no importance to either physician or patient to do so, as far as the immediate treatment of the fit is concerned. One, indeed, forms a clot after some time, which we have been just now discussing; and the other remains liquid. But that does not affect the question of the comatose condition now before us. I will speak, then, of "fluid," not distinguishing blood or serum, or bloody serum or serous blood, but clubbing them all, as must be done by practical men, together. When, then, there is fluid compressing the nerve-fibres, I feel sure that there is an influence for good in blood-letting, accompanied, of course, by a danger, as all active treatment is, but still a decided influence for good. It is capable of lessening the force of the heart, which is driving the blood towards the place where it is oozing out either as serum or complete blood, and of diminishing the calibre of the vessels that allow it to pass. The anxious question is, how to secure those advantages without the necessary accompanying dangers, without adding too great an additional shock to the already shocked nerves, without weakening fatally the already weakened general system.

You will observe, by the example of the patient I have used as my text, that the first effect of an apoplectic seizure is the violent blow to the nervous system above mentioned. The poor woman was damp and cold, and pale as a corpse; the pulse and heart beat quick, and irregularly, and weakly, just like those of an ox stunned by the slaughterer's pole-axe. Had she been bled then, she would certainly have died outright; and moreover, even had she survived, there was no object to be gained by it; for the heart was weak enough, surely. But after a time—seven hours in this instance, sometimes sooner, sometimes later—the heart and pulse recover, and the blood must be driven against the ruptured or oozing vessels with the same force which originally caused them to rupture or ooze. Now is the time to step in with the lancet. You prevent by it what often happens to apoplectic patients if left alone entirely; you prevent the relapse into coma which frequently follows an apparent partial recovery during reaction; you prevent it in a very intelligible way, by lowering the force of the blood-stream, which the injured vessels have already shown themselves unable to bear. This was the time, and this was the object, of the blood-letting in our patient's case. So complete was the apoplexy, by such a slender thread was she hanging on to life, that I believe the slightest additional extravasation of fluid in the cranium must have been fatal; and to this judicious use of the lancet by our house-surgeon, Mr. Chisholm, I attribute the patient's life.

You will observe the bleeding was not a large one—only eight ounces when reaction first occurred, and eight ounces again in the evening, when the pulse was again getting hard. I mention this to warn you against a mistake into which you might be led by an old and rather questionable maxim, "*Extremis morbis, extrema remedia.*" You might be disposed to say, the more the apoplexy, the more bleeding. Such a notion would be most dangerous. A small bleeding accomplishes the object in view; and a large one can do no more, while it seriously abridges the vital powers. Above all things, don't open the temporal artery; it is nearly equivalent to cutting your patient's throat.

I much prefer, in these cases, venesection to cutting the temples or the nape of the neck, as is sometimes done. What you want to do is to affect the general system, and particularly the centre of circulation—an object which is attained with most rapidity and certainty by opening a vein. You are aiming to prevent blood from being driven into the skull, not to extract that which is already in; at least, if you are striving after the latter result, your anatomy and physiology ought to have taught you better.

The effect of bleeding during the collapse, before reaction has taken place, is, as a rule, sudden death, especially when the heart or its valves are diseased. The effects of *over-bleeding* at a later period are, an excited condition of the circulation, and consequently a more violent impetus against the brain; and at the same time a more watery state of the blood, and consequently a greater tendency to serous effusion. As it is impossible to tell whether red blood or serum is exuded in the cranium, you run the greatest possible risk of augmenting the very evil which your injudicious zeal was intended to obviate.

Perhaps you may say, "I am going to bleed my patient again, for the sake of encouraging absorption; and surely absorption of the clot should be aimed at." Now, I am not so sure of that; if it were absorbed sooner than the nerve-fibres were ready to resume their functions, something else must be effused to take its place in the cranium. At all events, I am quite convinced that such absorption is best left to the reviving powers of nature; and you are diminishing these by unnecessarily depriving the body of blood.

In apoplexy and central paralysis, remember that you are dealing with diseases where nature still retains an inherent power of repair; in a moderately healthy constitution, the tendency is to get well. Do not, therefore, attribute all cases of recovery to the means used, unless you can give a probable physiological explanation of their beneficial action; but if you can thus rationally justify your treatment, do not hesitate to feel satified with it, whether successful or unsuccessful.

There is no necessity for being idle while you are awaiting the time to bleed. Stimulant injections and purgatives, hot water and mustard poultices to the feet and legs, a careful arrangement of the clothing and bedding so as to prevent congestion of the head, with keeping up the animal warmth and keeping the friends quiet, will amply occupy your time. It is also conducive to the end you have in view, to shave the head and apply cold lotions to the skull.

After reaction has returned, you will find advantage in the application of ice to the head; it is physiologically correct, and is often a great relief to the patient. You may remember that this poor woman used the first power of speaking that returned to her in begging me to continue the ice; whenever it was applied, it induced sleep, and was most agreeable. What a strange state a person must be in for ice to the head to be agreeable! But so it was.

When, then, you are called in to a case of apoplexy, let these considerations pass through your minds:

1—Does it depend on destructive softening of the cerebral substance? If so, I must not let anything persuade me to bleed.

2—Has the effusion taken place so long ago as to be, if blood, coagulated? If so, again, I had better abstain.

3—If it be fluid, bleeding is very much to be desired; it may prevent increase of the effusion, and relapse.

4—But to make bleeding most useful and least hurtful, the proper time must be selected, namely: the time when the heart regrains its strength.

5—The best guide is the circulation. The sharpness and hardness of the pulse and heart, as felt by you, are a faint picture of the sharpness and hardness of the pulse, as felt by the patient's brain. With your fingers on the wrist, let your mind travel into the interior of the skull.—*British Medical Journal, June* 8, 1861.

